# Nonlinear Optical Properties Tuning in *Meso*-Tetraphenylporphyrin Derivatives Substituted with Donor/Acceptor Groups in Picosecond and Nanosecond Regimes

**DOI:** 10.3390/molecules20045554

**Published:** 2015-03-27

**Authors:** Guanghong Ao, Zhengguo Xiao, Xuemin Qian, Zhongguo Li, Yuxiao Wang, Xueru Zhang, Yinglin Song

**Affiliations:** Department of Physics, Harbin Institute of Technology, Harbin 150001, China; E-Mails: aghoptics@163.com (G.A.); xiangyunonlinear@gmail.com (Z.X.); xmqian@hit.edu.cn (X.Q.); claes.lee@gmail.com (Z.L.); wangyx@hit.edu.cn (Y.W.)

**Keywords:** porphyrins, nonlinear refraction, nonlinear absorption, Z-scan, second hyperpolarizabilities

## Abstract

*meso*-Tetraphenylporphyrin (TPP) and its two substituted derivatives (*meso*-tetrakis(4-cyanophenyl)porphyrin [TPP(CN)_4_] and *meso*-tetrakis(4-methoxyphenyl)porphyrin [TPP(OMe)_4_]) were synthesized. Their nonlinear absorption and refraction properties were studied using the Z-scan technique in the picosecond (ps) and nanosecond (ns) regimes. The open aperture Z-scan results reveal that TPP and TPP(CN)_4_ display an identical reverse saturable absorption (RSA) character in the ps and ns regimes. While TPP(OMe)_4_ exhibits a transition from saturable absorption (SA) to RSA in the ps regime and a typical RSA character in the ns regime. The closed aperture Z-scan results show that TPP(CN)_4_ and TPP(OMe)_4_ have regular enhancement of the magnitude of nonlinear refraction as compared to their parent TPP in both the ps and ns regimes. In addition, the second-order molecular hyperpolarizabilities (*γ*) of these three porphyrins are calculated, and the *γ* values of TPP(CN)_4_ and TPP(OMe)_4_ are remarkable larger than that of TPP. The introduction of the electron-withdrawing group CN and the electron-donating group OMe into TPP has enhanced its nonlinear refraction and *γ* value, and tuned its nonlinear absorption (TPP(OMe)_4_), which could be useful for porphyrin-related applications based on the desired NLO properties.

## 1. Introduction

Organic materials such as polymethine dyes [1], chromophores [2], phthalocyanines [3], porphyrins [4], and their related compounds [5] are target materials to study nonlinear optical (NLO) properties. The conjugated π-electron bonding networks in these molecules are the principal reason for their typical NLO properties [6,7]. Recent years the centrosymmetric or asymmetric structure, non-substituted or substituted configuration, and the metal derivatives of these organic compounds have been commonly studied [8,9]. Porphyrins are one of the attractive organic compounds with large nonlinear optical (NLO) properties [10,11,12,13,14]. Moreover, the electronic structure of porphyrins can flexibly be tailored by the variation of peripheral substituents or the ligands of central atoms for tuning their NLO properties [5,15]. Recent years, significant efforts have been devoted to the establishment of structure-NLO properties relationships for porphyrin-based molecules so that materials with improved and optimized NLO properties can be rationally designed and synthesized for particular applications. As expected, substantial progresses have been achieved, especially for the optical limiting (OL) property in the form of reverse saturable absorption (RSA) [5,8,16,17,18,19,20]. It was concluded that the enhancement of OL behavior can be indeed achieved by the variation of peripheral substituents, the extension of π-conjugation and the ligand of central atom [9,21,22]. In addition, saturable absorption (SA) in porphyrin-based compounds was also observed, which are mainly dependent on excitation wavelength, excitation intensity, concentration and molecular structure [23,24,25].

Aside from the referred nonlinear absorption of RSA and SA, nonlinear refraction is also an important NLO process, which can be caused by nonlinear absorption *via* causality and Kramers-Kronig relations [26]. As nonlinear refraction could lead to a modulation of the phase or amplitude of an intense optical wave in a nonlinear medium, hence it has attracts significant scientific and technological interest for its various applications related to optical limiting, nonlinear spectroscopy, optical switching and optical communications and so on [27,28]. On the other hand, nonlinear refractive index may result in unwanted phase distortion or even optical damage, which should be taken well consideration in the high power beam related experiments and applications. Therefore, the investigation of nonlinear refraction properties is also of great significance for particular applications. Until now, although the nonlinear refraction properties of some porphyrin-related molecules were reported [21,22,29,30,31,32,33,34,35], the reports on nonlinear refraction of prophyrins are still sparse.

**Figure 1:**
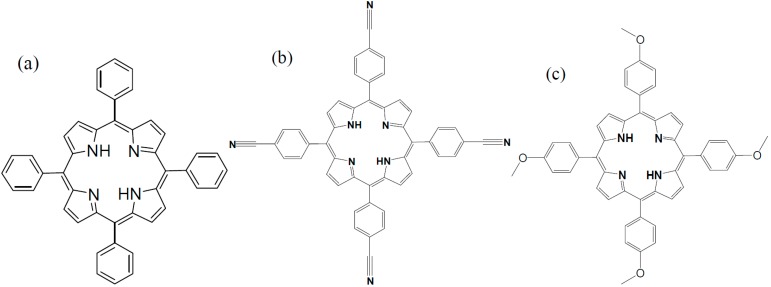
The molecular structures of (**a**) *meso*-tetraphenylporphyrin (TPP); (**b**) TPP(CN)_4_ and (**c**) TPP(OMe)_4_.

The Z-scan technique is a simple and effective method to study the nonlinear absorption and refraction properties of nonlinear media [36], which has been widely used in ns, ps, and fs regimes [12,22,29]. In this paper, we reported our recent finding on the nonlinear absorption and refraction properties of *meso*-tetraphenylporphyrin (TPP) and its two substituted derivatives (*meso*-tetrakis(4-cyanophenyl)porphyrin [TPP(CN)_4_] and *meso*-tetrakis(4-methoxyphenyl)porphyrin [TPP(OMe)_4_]) using the Z-scan technique in the picosecond (ps) and nanosecond (ns) regimes. Furthermore, the second molecular hyperpolarizabilities of these three porphyrins are calculated. The molecular structures of TPP, TPP(CN)_4_ and TPP(OMe)_4_ are shown in Figure 1.

## 2. Results and Discussion

### 2.1. Linear Absorption Characterization

**Figure 2:**
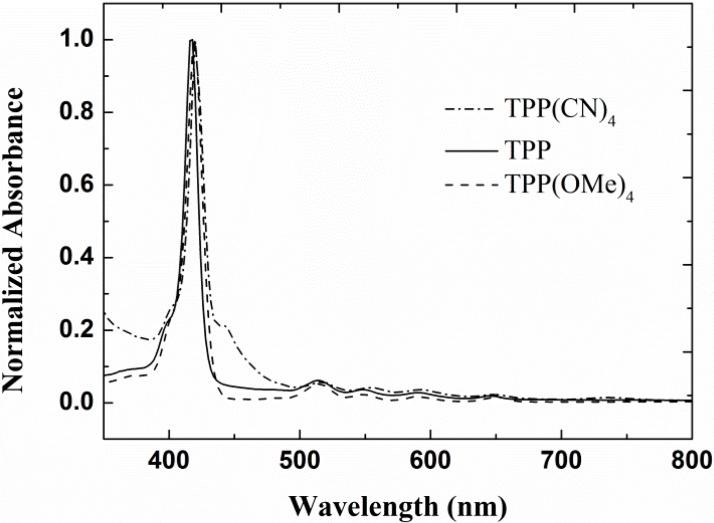
UV-visible absorption spectra of TPP, TPP(CN)_4_, and TPP(OMe)_4_ in CH_2_Cl_2_.

The UV-visible spectra of TPP, TPP(CN)_4_, and TPP(OMe)_4_ in CH_2_Cl_2_ are presented in Figure 2. The Soret band of these compounds is centered around 420 nm, and the Q-bands are all located between 500 and 700 nm [12]. It can be seen from Figure 2 that the incorporation of the donor methoxy group and acceptor cyano group into TPP did not arouse obvious change in its linear absorption spectra.

### 2.2. Picosecond Z-Scan Measurements

**Figure 3:**
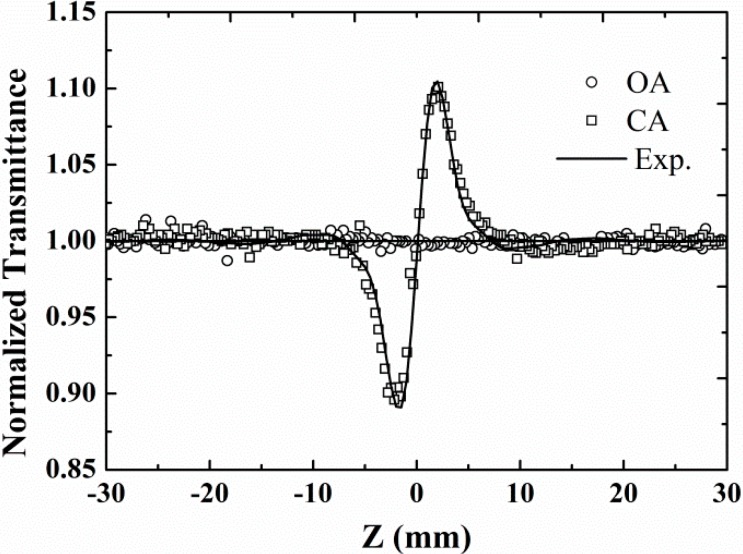
Open aperture (OA) and closed aperture (CA) Z-scan data of CH_2_Cl_2_ with the same on-axis intensity 1.1 GW/cm^2^.

To check the effect of CH_2_Cl_2_ solvent on the nonlinear absorption and refraction of the entire porphyrin/CH_2_Cl_2_ solution, the Z-scan measurements are first performed on CH_2_Cl_2_, as shown in Figure 3. The circles show the normalized transmission without an aperture (open aperture, OA), which is varying as a function of distance along the lens axis. The result reveals that no nonlinear absorption of CH_2_Cl_2_ could be observed within the limit of the intensity (1.1 GW/cm^2^) used in the experiment. However, the closed aperture (CA) result (squares) shows an obvious valley-peak configuration, which indicates a positive nonlinear refraction.

**Figure 4:**
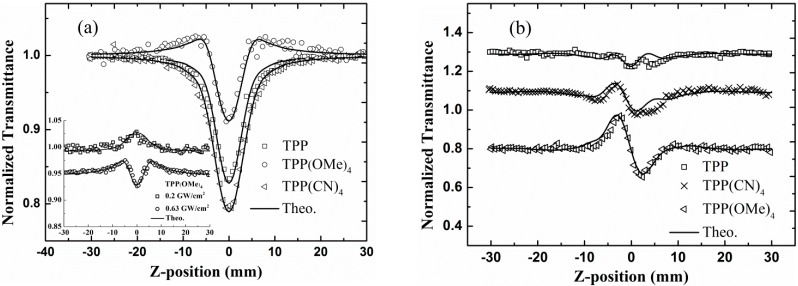
Open (**a**) and closed aperture (**b**) Z-scan data of TPP, TPP(CN)_4_, and TPP(OMe)_4_ with 21 ps pulses, on-axis intensity of 1.1 GW/cm^2^. Solid lines are theoretical fits. Inset shows OA Z-scan data at 0.2 and 0.63 GW/cm^2^, respectively.

The Z-scan measurements along with the corresponding fits for TPP, TPP(CN)_4_, and TPP(OMe)_4_ in CH_2_Cl_2_ are shown in Figure 4. Figure 4a presents the OA Z-scan results. TPP and TPP(CN)_4_ both exhibit a notable valley at the focus, indicating a reverse saturable absorption (RSA) character with a positive nonlinear absorption coefficient. Whereas TPP(OMe)_4_ shows a different absorption property. It experiences a switch from saturable absorption (SA) at a low input intensity to RSA at higher intensities. The inset in Figure 4a clearly provides the SA pattern of TPP(OMe)_4_ at the input intensity 0.2 GW/cm^2^, and the coexistence of SA and RSA at 0.63 GW/cm^2^. It can be concluded that RSA effect will play a dominant role as the input intensity increasing largely enough. In consideration of the negligible nonlinear absorption of CH_2_Cl_2_ (in Figure 3), the nonlinear absorption effects in Figure 4a can be mainly attributed to the solutes rather than CH_2_Cl_2_ solvent. As mentioned above, the occurrence of SA heavily depends on excitation wavelength, excitation intensity, concentration and molecular structure [18,19,20], and the first three parameters for all the compounds are same in our experiment, thus we attributed the SA-to-RSA behavior in TPP(OMe)_4_ to its unique molecular structure. This implies that the incorporation of electron-donating groups (methoxy) into TPP can turn its nonlinear absorption property.

The OA and CA Z-scan curves are fitted based on the coupled equations involving the irradiance and the nonlinear phase shift [36]. Furthermore, the nonlinear absorption switching behavior of TPP(OMe)_4_ is described by a nonlinear absorption coefficient of $\text{α }\left(I\right)\text{= }\frac{{\text{α}}_{0}}{1\text{+}I/I_{\text{s}}}{\text{+β}}_{\text{eff}}I$ [37]. Here, α_0_ is the linear absorption coefficient of the sample. β_eff_ is the effective nonlinear absorption coefficient and *I*_s_ is the saturable intensity. For the RSA behavior in TPP and TPP(CN)_4_, the saturable intensity *I*_s_ → ∞ and hence the nonlinear absorption coefficient can be simplified to α(*I*) = α_0_ + β_eff_*I*. The fittings in Figure 4a with such nonlinear absorption coefficients yield β_eff_ values for TPP, TPP(CN)_4_, and TPP(OMe)_4_, which are 0.65, 1.0, and 4.0 cm/GW, respectively. Furthermore, the saturable intensity *I*_s_ of TPP(OMe)_4_ can be obtained by fitting and is 1.25 ± 0.25 GW/cm^2^. 

The CA Z-scan curves (Figure 4b) for the three porphyrins in CH_2_Cl_2_ exhibit primary peak-valley configurations. Since the CA Z-scan result of CH_2_Cl_2_ solvent has a valley-peak configuration (Figure 3), it is obvious that the presence of the curves in Figure 4b is attributed to the combined effect of porphyrins solute and CH_2_Cl_2_ solvent. Furthermore, the solvent contribution has reduced the actual magnitude of nonlinear refraction in these porphyrins due to the opposite signs of their nonlinear refraction. Therefore, all the measured porphyrins have a negative nonlinear refractive index. In addition, it is clear in Figure 4b that TPP(OMe)_4_ has the largest difference between the normalized transmittance at peak and valley positions. This implies a larger nonlinear refraction index of TPP(OMe)_4_ than those of TPP and TPP(CN)_4_. While TPP with the smallest magnitude of normalized transmittance holds a comparative absolute value of nonlinear refraction index compared with that of CH_2_Cl_2_. The theoretical curves are obtained from the best fits with the change in refractive index expressed as $\Delta n=n_{2\text{eff}}I$ , where, *n*_2eff_ is the effective nonlinear refractive index. The curves are reasonably well matches with the experimental data. The true *n*_2eff_ values for these molecules are extracted dependent on the experimental data in Figure 3 and Figure 4b, which are −1.9, −3.2 and −9.6 (×10^−6^ cm^2^/GW) for TPP, TPP(CN)_4_, and TPP(OMe)_4_, respectively. TPP(CN)_4_ and TPP(OMe)_4_ have notable nonlinear refraction compared to their parent system TPP. Moreover, the nonlinear refraction of TPP(OMe)_4_ has a more remarkable enhancement.

### 2.3. Nanosecond Z-Scan Measurements

**Figure 5:**
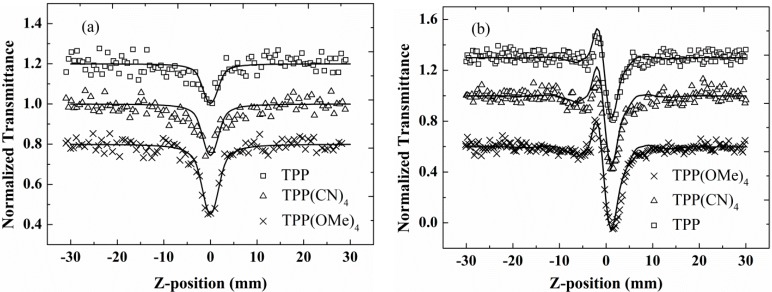
Open (**a**) and closed aperture (**b**) Z-scan data of TPP, TPP(CN)_4_, and TPP(OMe)_4_ with 6 ns pulses, on-axis intensity 27 MW/cm^2^. Solid lines are theoretical fits.

To examine the nonlinear properties of these porphyrins in nanosecond regime, we performed the Z-scan measurements with 6 ns laser pulses. Figure 5a gives the open aperture Z-scan curves. Each of the compounds displays RSA characters under the excitation of nanosecond pulses. The CA curves of these three porphyrins are all characterized by peak-valley configurations (Figure 5b), which indicate the negative nonlinear refractions due to the coexistence of electronic effect and thermal-lensing effect [38]. The buildup time of thermal lensing can be determined by *ω*_0_/*υ*_s_ [39]. *ω*_0_ is the laser beam radius at focus and it is ~19 μm in our Z-scan with 6 ns laser pulses. The velocity of sound *υ*_s_ in CH_2_Cl_2_is 1090 m/s [39]. Hence, the calculated buildup time of thermal nonlinearity in our experiment is ~17.4 ns. Although it is longer than the pulse duration we used, the thermal lensing effect may even come into play, which belongs to the problem of the thermally induced optical nonlinearities in liquids in the transient regime [40]. Since the nonlinear absorption and refraction of the solvent under the identical experimental conditions are extremely weak, the both of them could be neglected in the ns regime.

In the thin sample and slowly varying envelope approximations, the beam propagating equations separately governing the irradiance (*I*) and the phase (*ϕ*) are:

(1) $$\begin{array}{l} \frac{\text{d}I}{\text{d}z^{\prime }}=-\left({\text{α}}_{0}+\beta_{\text{eff}}I\right) \end{array}$$

(2) $$\frac{\text{d}\phi }{\text{d}z^{\prime }}=k\left(n_{2\text{eff}}I+\Delta n_{\text{th}}\right)$$

Here, *z'* is the propagation length in the sample. Parameters α_0_ and β_eff_ denote the linear and nonlinear absorption coefficients, respectively. *k* is the wavevector, and *n*_2_ is the nonlinear refraction coefficient. Δ*n*_th_ is the transient thermal index change induced by the heating of the solution as a result of the absorption of laser pulses. It is obtained by solving the photoacoustic wave equation [38,40], and it is expressed as Equation (3) (see [38,40]).

(3) $$\frac{\partial^{2}\Delta n_{\text{th}}}{\partial t^{2}}-\upsilon_{s}^{2}\nabla^{2}\left(\Delta n_{\text{th}}\right)=\left(\text{d}n/\text{d}T\right)\upsilon_{\text{s}}^{2}\nabla^{2}\left(\Delta T\right)$$

Herein, d*n*/d*T* is the rate of change of the index with respect to temperature and Δ*T* is the temperature change in the sample induced by the absorption of laser radiation, which is described by:

(4) $$\Delta T\text{ = }\frac{1}{{\text{ρ}}_{0}C_{p}}\int_{-\infty }^{t}\left({\text{α}}_{0}+{\text{β}}_{\text{eff}}I\right)I\text{d}t$$

The related parameters in the simulations are taken from Ref. [39], which are *υ*_s_ = 1090 m/s, d*n*/d*T* = 5.5 × 10^−4^ K^−1^, *C*_p_ = 1.6 × 10^3^ J/(cm^3^ K), respectively. The density of CH_2_Cl_2_ is ρ_0_ = 1.33 × 10^3^ kg/m^3^. Based on the theory and the related parameters above, the nonlinear coefficients of these molecules could be extracted, which are 100, 150 and 360 cm/GW for TPP, TPP(CN)_4_, and TPP(OMe)_4_, respectively. Meanwhile the nonlinear refractive index of TPP, TPP(CN)_4_, and TPP(OMe)_4_ are −1.3, −1.7 and −4.6 (×10^−3^ cm^2^/GW), respectively. Note that the electronic effect plays a dominant role in the nonlinear refraction of these three porphyrins. It can be seen that the electron-withdrawing substituent CN and electron-donating substituent OMe both have led to the enhancement of nonlinear refraction in the ps and ns regimes, whereas their effects on the nonlinear absorption property are weak as compared with that of TPP due to the different extent of linear absorption, except that TPP(OMe)_4_ has a SA-to-RSA switch in the case of ps pulses.

### 2.4. Second-Order Molecular Hyperpolarizabilities

As controlling of the second-order molecular hyperpolarizabilities (*γ*) is crucial for many applications in electronic and photonic devices, we calculated the *γ* values of the porphyrins investigated by the formulation Equation (5) [41]:

(5) $$\gamma \text{ = }\frac{\chi^{\left(3\right)}}{N_{\text{c}}L}$$

where *N*_c_ is the molecular number density per cubic centimeter, *L* is the local-field correction factor, which is approximately to be ${\left(\left(n_{0}^{2}\text{+}2\right)/3\right)}^{4}$ . *χ*^(3)^ is the nonlinear susceptibility, which can be obtained by the real (Re *χ*^(3)^) and imaginary (lm*χ*^(3)^) parts of it by:

(6) Reχ(3) = cn02120πn2eff

(7) $$\text{lm}\chi^{\left(3\right)}\text{ = }\frac{c^{2}n_{0}^{2}}{240\pi \omega }{\text{β}}_{\text{eff}}$$

Here, *c* is the speed of light, *n*_0_ is the linear refractive index of the sample, and *ω* is the angular frequency of the laser pulses. *n*_eff_ and β_eff_ have been extracted above. In the picosecond regime, the *γ* values of TPP, TPP(CN)_4_, and TPP(OMe)_4_ are 1.1 × 10^−30^, 1.7 × 10^−30^, and 5.8 × 10^−30^ esu, respectively. Both TPP(CN)_4_ and TPP(OMe)_4_ have an enhanced *γ* value as compared to that of TPP. While in the nanosecond regime, the *γ* values for TPP, TPP(CN)_4_, and TPP(OMe)_4_ are 441, 585, and 1563 (×10^−30^ esu), respectively. 

Recently, the second-order molecular hyperpolarizabilities of some porphyrin-related compounds in picosecond regime have been reported. Venugopal Rao *et al.* [42] have reported *γ* values at 532 nm, 35 ps from the DFWM measurements of sixteen porphyrins with different metal ions in the ring. The highest value of 1.432 × 10^−30^ esu was observed for CoTPP. The difference of nonlinear optical nonlinearities between the porphyrins is attributed to the higher electron delocalization in the molecules. Chen *et al.* [41] have reported the second molecular hyperpolarizabilities of a azobenzene-phthalocyanine dyad measured by a Z-scan technique at 532 nm with a pulse duration of 25 ps. which are 3.87 × 10^−30^ and 4.82 × 10^−30^ esu before and after illumination with 365 nm UV light, respectively. Shi *et al.* [30] reported the second hyperpolarizabilities of four porphyrin-heteropolyoxometalate hybrid system and TPP, which are characterized by Z-scan measurements at 532 nm with a pulse duration of 20 ps. The *γ* value they calculated for TPP is 0.84 × 10^−30^ esu. The other four compounds possess *γ* values between 0.49−1.2 × 10^−30^ esu. Obviously, the *γ* value of TPP(OMe)_4_ in our study is remarkable larger than those of the related compounds mentioned above in the ps regime. The obtained results imply that the introduction of the cyano or methoxy groups into TPP has obviously tuned its refraction and absorption properties, leads to the enhancements of the nonlinear refraction and the second molecular hyperpolarizabilities, which is expected to provide useful reference for exploring porphyrin derivatives with much higher second hyperpolarizabilities [43].

## 3. Experimental Section

5,10,15,20-Tetraphenylporphyrin (TPP), 5,10,15,20-tetrakis(4-cyanophenyl)porphyrin [TPP(CN)_4_] and 5,10,15,20-tetrakis(4-methoxyphenyl)porphyrin [TPP(OMe)4] were synthesized following the method used by Capitosti *et al* [44]. 

The nonlinear absorption and nonlinear refraction of TPP, TPP(CN)_4_ and TPP(OMe)_4_ were measured by Z-scan techniques in ps and ns regime. The light source of 21 ps (FWHM) pulses is generated from a Q-switched Nd: YAG laser (PL2143A, EKSPLA, Vilnius, Lithuania) with a wavelength of 532 nm. The laser system for nanosecond Z-scan was a Surelite Q-switched Nd:YAG 532 nm laser (Continuum, Santa Clara, CA, USA) with a pulse width of 6 ns (FWHM). The spatial profiles of both ps and ns pulses have a top-hat distribution. The Z-scan techniques were arranged as that reported by Yang *et al.* [45]. The beam waist at the focus is ~26 μm for ps and ~19 μm for ns, respectively. The porphyrins are dissolved in dichloromethane (CH_2_Cl_2_) with the same concentration of 8.14 × 10^−5^ M and placed in 2 mm-thickness quartz cells, the corresponding linear absorption coefficients are 0.8, 1.1, and 5.8 cm^−1^ for TPP, TPP(CN)_4_, and TPP(OMe)_4_. The incident and transmitted pulses energies were measured simultaneously by the same type energy detectors (RjP-765a, Laser Probe Inc., Utica, NY, USA). The energy meter we used is a Laser Probe Inc., Rj-7620 ENERGY RATIOMETER.

## 4. Conclusions

The nonlinear absorption and nonlinear refraction properties of TPP, TPP(CN)_4_, and TPP(OMe)_4_ are measured by the Z-scan technique at 532 nm in the ps and ns regimes. TPP and TPP(CN)_4_ display an identical reverse saturable absorption (RSA) character in the ps and ns regimes. While TPP(OMe)_4_ exhibits a transition from saturable absorption (SA) to RSA in the ps regime, and a typical RSA character in the ns regime. Furthermore, TPP(CN)_4_ and TPP(OMe)_4_ show regular enhancement of the magnitude of nonlinear refraction compared to their parent TPP in both the ps and ns regimes. The obtained results related to the three porphyrins in both the ps and ns regimes suggest that the introduction of the cyan or methoxy group into TPP has obviously tuned its NLO properties, leads to the enhancements of the nonlinear refraction and the second molecular hyperpolarizabilities, which is useful for the development of porphyrin-related applications upon desired nonlinear optical properties.
